# Simulation of facial expressions using person-specific sEMG signals controlling a biomechanical face model

**DOI:** 10.1007/s11548-017-1659-5

**Published:** 2017-08-31

**Authors:** Merijn Eskes, Alfons J. M. Balm, Maarten J. A. van Alphen, Ludi E. Smeele, Ian Stavness, Ferdinand van der Heijden

**Affiliations:** 1 Department of Head and Neck Oncology and Surgery, Netherlands Cancer Institute, Plesmanlaan 121, 1066 CX Amsterdam, The Netherlands; 2 MIRA Institute of Biomedical Engineering and Technical Medicine, University of Twente, Drienerlolaan 5, 7522 NB Enschede, The Netherlands; 3 Department of Oral and Maxillofacial Surgery, Academic Medical Center, Meibergdreef 9, 1105 AZ Amsterdam, The Netherlands; 4 Department of Computer Science, University of Saskatchewan, 176 Thorvaldson Building, 110 Science Place, Saskatoon, SK S7N 5C9 Canada; 5 P.O. Box 90203, 1006 BE Amsterdam, The Netherlands

**Keywords:** Forward modelling, Biomechanical modelling, Surface electromyography, Lips, Head and neck cancer, Functional inoperability

## Abstract

**Purpose:**

Functional inoperability in advanced oral cancer is difficult to assess preoperatively. To assess functions of lips and tongue, biomechanical models are required. Apart from adjusting generic models to individual anatomy, muscle activation patterns (MAPs) driving patient-specific functional movements are necessary to predict remaining functional outcome. We aim to evaluate how volunteer-specific MAPs derived from surface electromyographic (sEMG) signals control a biomechanical face model.

**Methods:**

Muscle activity of seven facial muscles in six volunteers was measured bilaterally with sEMG. A triple camera set-up recorded 3D lip movement. The generic face model in ArtiSynth was adapted to our needs. We controlled the model using the volunteer-specific MAPs. Three activation strategies were tested: activating all muscles $(\hbox {act}_\mathrm{all})$, selecting the three muscles showing highest muscle activity bilaterally $(\hbox {act}_3)$—this was calculated by taking the mean of left and right muscles and then selecting the three with highest variance—and activating the muscles considered most relevant per instruction $(\hbox {act}_\mathrm{rel})$, bilaterally. The model’s lip movement was compared to the actual lip movement performed by the volunteers, using 3D correlation coefficients $(\rho )$.

**Results:**

The correlation coefficient between simulations and measurements with $\hbox {act}_\mathrm{rel}$ resulted in a median $\rho$ of 0.77. $\hbox {act}_3$ had a median $\rho$ of 0.78, whereas with $\hbox {act}_\mathrm{all}$ the median $\rho$ decreased to 0.45.

**Conclusion:**

We demonstrated that MAPs derived from noninvasive sEMG measurements can control movement of the lips in a generic finite element face model with a median $\rho$ of 0.78. Ultimately, this is important to show the patient-specific residual movement using the patient’s own MAPs. When the required treatment tools and personalisation techniques for geometry and anatomy become available, this may enable surgeons to test the functional results of wedge excisions for lip cancer in a virtual environment and to weigh surgery versus organ-sparing radiotherapy or photodynamic therapy.

**Electronic supplementary material:**

The online version of this article (doi:10.1007/s11548-017-1659-5) contains supplementary material, which is available to authorized users.

## Introduction

Surgical treatment in advanced head and neck cancer can lead to severe function loss, including chewing deficits, dysphagia, and speech impairment. If this function loss is expected to be unacceptable, then other treatments, like radiotherapy, chemotherapy and photodynamic therapy, can be considered [1]. Although alternative curative treatments like radiotherapy have their own effects on functional outcome, in the future we will focus on surgical effects first by developing a virtual surgery tool because these are relatively easier to model. Unfortunately, it is difficult to predict functional outcome of the aforementioned treatments accurately. In fact, the prediction depends heavily on the subjective judgements by members of the multidisciplinary tumour board and therefore can differ greatly among specialists [2]. To tackle the problem of preoperative prediction of surgical outcome, biomechanical models are preferred as these models can be adjusted to represent the actual anatomy and pathological anatomical changes and they can simulate physical processes. Biomechanical models of the head and neck region have been developed. In particular, in the field of animation and facial surgery planning [3–7], these models mainly predicted aesthetic outcome. A couple of those models also predicted functional outcome, like effects of scar tissue on tongue mobility [8, 9], intraoral swallowing effects [10], and facial expressions after maxillofacial surgery [11]. The models can be controlled by simulated muscle activation patterns. These activation patterns contract the models’ muscles (elements) resulting in a visible movement. This process is called forward modelling: the determination of motion calculated from known forces. Each person learns to perform functional tasks (e.g. mastication, speech, and swallowing) with a specific motor control strategy. These strategies differ per person. Moreover, muscular compensatory mechanisms might be used after impairment. Forward modelling is a prerequisite for prediction of functional consequences after surgery using biomechanical models. When a tumour is virtually resected in a model, forward modelling may give insight in residual movement when controlling the adapted model with the patient’s muscle activation strategies, whereas inverse modelling (calculating the required muscle activation patterns from known movement) may give insight in compensatory possibilities. This residual movement can then be addressed by the multidisciplinary medical team, and function loss may be estimated. Modelling of the perioral region is of interest because it is easily accessible and can serve as a proof of principle for more complex organs like the tongue. Besides, surgery of the lips could lead to both cosmetic and function deficits. Lip cancers are surgically treated by wedge excision and primary closure with consequences for pursing the lips and opening the mouth. Larger defects require reconstruction with local or free flaps. In those last cases, function preservation is even more at stake. The obtained information on function loss with the use of our future models may deliver patient and physician an overview of the possible cosmetic and function deficits of the different treatment options, both surgical and nonsurgical curative alternatives such as radiotherapy or photodynamic therapy. This also increases the quality of patient counselling. In other words, it makes objective and more informed choices in treatment options possible.

Biomechanical models have been created for many parts of the human body: upper and lower limb, shoulder, elbow, and wrist (see Erdemir et al. [12] for an overview). These models are commonly used for calculating forces on regions of interest. The models are controlled by muscle activation patterns, which can also be derived from electromyographic (EMG) signals. Research in EMG-controlled biomechanical models of the perioral region is still a subject of ongoing research [13–18]. Lucero and Munhall [18] used intramuscular EMG as input for standard Hill-type muscles in their biomechanical face model with a multilayer deformable mesh. Although reasonably good correlation coefficients were obtained between the model’s movement and measured movement in general, markers around the lips performed poor in anterior–posterior direction. Flynn et al. [17] developed a model of the face with a complex anisotropic multilayer skin with in vivo tension. They, and others, tested their model by manually activating facial muscles to obtain simulated facial expressions [13, 19, 20].

Before embarking on complex personalised biomechanical models including the patient’s as well as the tumour’s geometry and anatomy, we investigated in this study whether we could supply such models with patient-specific motor control by means of sEMG measurements.

**Fig. 1:**
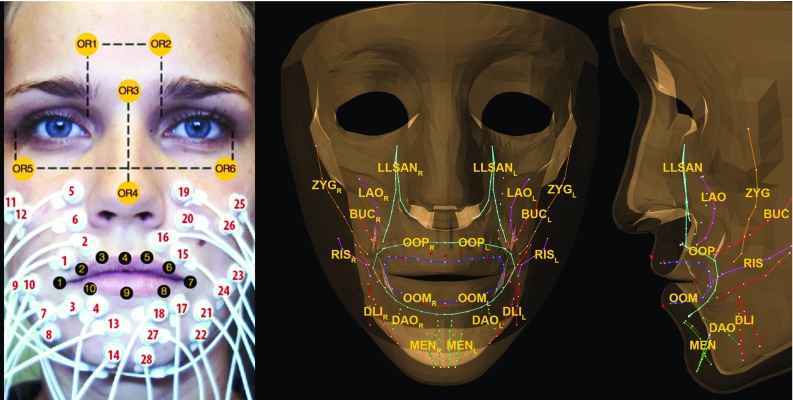
*Left* Surface electrode locations, orientation markers, and lip markers. *Right* Anterior–posterior view and lateral view of the model and the model’s muscle bundles and lip markers. The muscles are abbreviated as follows: zygomaticus major (ZYG), risorius (RIS), levator labii superioris alaeque nasi (LLSAN), levator anguli oris (LAO), buccinator (BUC), orbicularis oris peripheralis (OOP) and marginalis (OOM), depressor labii inferior (DLI), depressor anguli oris (DAO), and mentalis (MEN), subscript L for ten left-sided muscles and subscript R for ten right-sided muscles

In previous research we demonstrated that noninvasive surface EMG (sEMG) conveys sufficient information to predict static facial expressions and volunteer-specific lip motion [21, 22]. However, these statistical models lack the physiological relationship required to predict surgical outcomes. Therefore, we aim to demonstrate that a biomechanical 3D lip model can be controlled by muscle activation patterns derived from volunteer-specific sEMG signals of facial muscles to simulate facial expressions. These principles will be applicable in our future projects in which we will add a virtual surgery tool and in which we plan to take the step towards intraoral sEMG measurements of the tongue muscles to make tongue models more patient specific. The results demonstrated in this study may not only be of interest in speech research or in facial animation but also in biomechanics research with an important prelude for our virtual surgery models. Demonstrating the feasibility of driving biomechanical face models via individual sEMG measurements is important because it forms the basis for affected function by patient-specific motor control. When a virtual surgery tool or radiotherapy tool becomes available, it will show the movement in the affected situation based on personalised innervation signals.

## Methods

### Volunteers and data acquisition

For detailed information regarding the data acquisition, we refer to Eskes et al. [22]. Here follows a summary: six healthy volunteers participated, three males and three females, ages ranging from 21 to 30. We measured sEMG signals ($s_m$, signal per muscle channel *m*) with the TMSi$^\circledR$ Porti$^{\mathrm{TM}}$ system (TMSi$^\circledR$, Oldenzaal, the Netherlands) of seven facial muscles bilaterally (see Fig. 1). A common ground self-adhesive reference electrode was placed on the left wrist. Six optical face markers—for head orientation—and ten optical lip markers—to follow lip movement—were drawn using a skin marker (Fig. 1) and tracked at 100 frames per second with our triple camera set-up [22, 23]. The Medical Research Ethics Committee of the Netherlands Cancer Institute approved this study, and the volunteers gave their informed consent.

### Instructions to volunteers

**Fig. 2:**
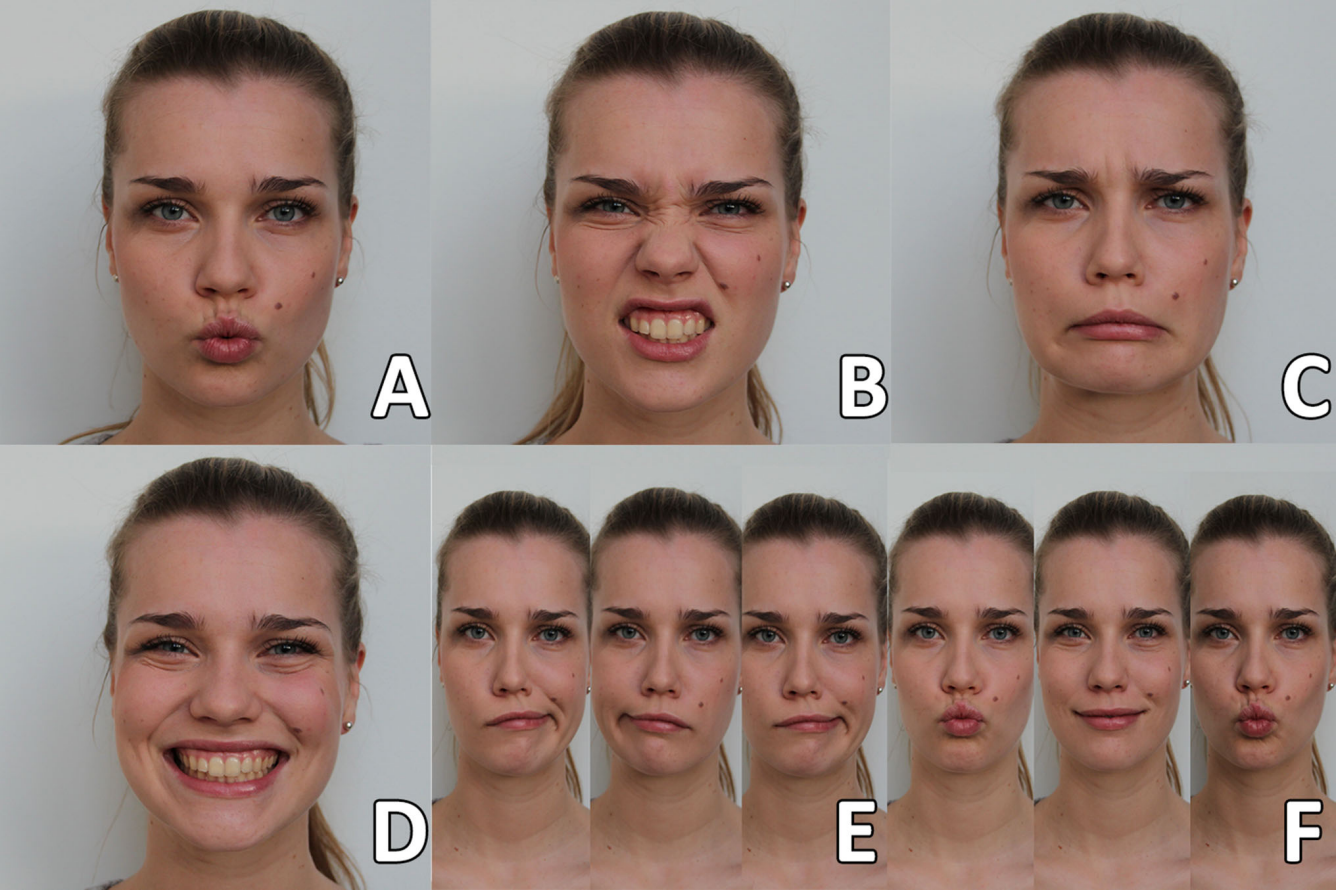
Instructions to volunteers: **A** purse lips, **B** raise upper lip, **C** depress mouth corners, **D** voluntary smile, **E** left-right-left with closed mouth, **F** purse lips–closed mouth smile–purse lips

Volunteers performed four facial expressions to maximise independent muscle contraction of the recorded muscles: (A) purse lips, (B) raise upper lip, (C) depress mouth corners, and (D) voluntary smile, an asymmetric motion: (E) left-right-left with closed lips, and a combination of two expressions: (F) purse lips and closed mouth smile (Fig. 2). These facial expressions were based on the work of Lapatki et al. [24] and Schumann et al. [25]. At the start of the experiment the instructions were shown to the volunteer in combination with a live demonstration by the experimenter. Oral feedback on the volunteer’s performance was given, while he or she was repeating the instructions four times with 2-s rest in between.

### Finite element face model

We used the reference finite element face model in ArtiSynth that was originally developed with ANSYS$^\circledR$ software at the ICP/GIPSA and TIMC-IMAG laboratories in Grenoble [15, 16, 26–28]. It is described in detail in Nazari et al. [14]. The most important details are as follows. The soft tissues of the face are represented by three layers of elements and includes 6342 elements (6024 linear hexahedral and 318 linear wedge) and 8720 nodes. The epidermis and dermis are contained in the outer layer of about 1.5 mm thick. The hypodermis comprises the inner and centre layers that vary between 4 and 11 mm in thickness. All layers were given the same passive tissue properties, including tissue density of $1040\;\hbox {kg/m}^{{3}}$, and material stiffness specified as a Mooney–Rivlin constitutive equation given by:

1 $$\begin{aligned} W=C_{10} (\tilde{I}_1 -3)+C_{20} (\tilde{I}_1 -3)^{2}+\frac{\kappa }{2}(\ln J)^{2} \end{aligned}$$

where *W* is the stress energy, and $C_{10} =2.5\,\hbox {kPa}$, $C_{20} =1.175\,\hbox {kPa}$, and $\kappa =25\,\hbox {kPa}$ are the material parameters. The left Cauchy–Green tensor: ${\tilde{\mathbf{B}}}=\tilde{\mathbf{F}}\tilde{\mathbf{F}}^\mathrm{T}$ is used to calculate $\tilde{I}_1 =\hbox {trace}(\tilde{\mathbf{B}})$, and $J=\det (\mathbf{F})$. The distortional part of the deformation gradient $\mathbf{F}$ is described by $\tilde{\mathbf{F}}=j^{-1/3}{} \mathbf{F}$.

The facial muscles were represented by muscle fibres within the finite element mesh, and they are organised into 20 muscle groups (Fig. 1). During simulations finite element muscles were used in which the elements surrounding the fibres were assigned as muscle elements with transversely isotropic material properties described by Blemker et al. [29]. Elements that were within a radius of 5 mm of the muscle fibres were considered a muscle element. In the case of the orbicularis oris peripheralis (OOP) and marginalis (OOM) muscle elements were manually assigned.

Common muscle model parameters were used across volunteers. They are based on values from the literature [17]: maximum stretch ${\lambda }^{*}=1.4$, where the force–stretch relationship becomes linear, exponential stress coefficient $P_1 =0.05$, and uncrimping factor $P_2 =6.6$. The maximum stress of the muscle elements $\sigma _{\max }$, as exception, was optimised per volunteer by decreasing the maximum stress with 10% each time inverted elements occurred, starting at $300\,\hbox {kPa}$.

The mandible and maxilla underlying the face tissue model were represented as rigid bodies. Gravity acted on the model with acceleration set to $-9.8\,\hbox {m/s}^{2}$ in vertical direction.

### Boundary conditions, collision behaviour, and incompressibility

**Fig. 3:**
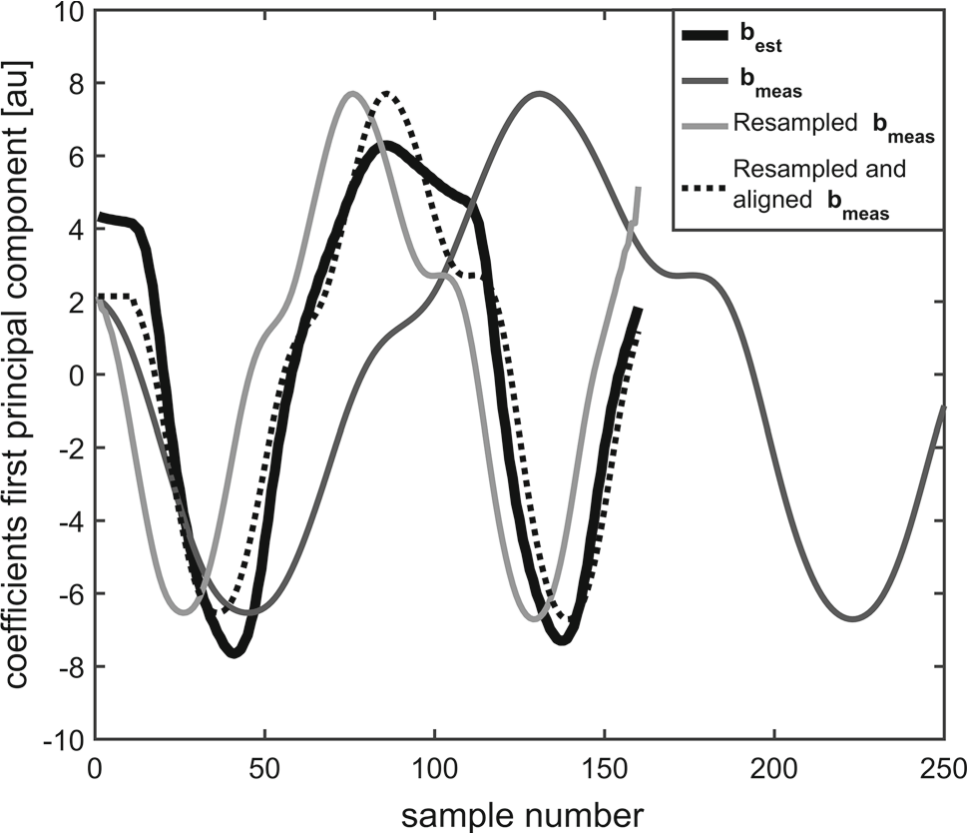
Synchronisation example. The first principal component coefficient vectors of the measurement $\mathbf{b}_\mathrm{meas}$ and the model output $\mathbf{b}_\mathrm{est}$ are shown. Together with the resampled coefficient vector, and the resampled and aligned coefficient vector

Nodes on the inner surface of the finite element face model were attached to the underlying mandible and maxilla (similar to the attachments shown in Stavness et al. [28]; Fig. 3). The nodes of the centre and the outer layer were dynamic. Contact of elements, which is especially important when pressing the lips together, is handled with the mesh-based collision behaviour in ArtiSynth. Interpenetration of the upper and lower lip surfaces is detected; node penetrations are corrected with impulse-based contact constraints [30]. The friction coefficient for contacts was set to zero. Instead of constraint-based soft tissue incompressibility, we used nodal soft incompressibility with a quadratic bulk potential and a bulk modulus of $25\,\hbox {kPa}$. Soft incompressibility tries to ensure that the volume of the finite element model remains locally constant by generating a restoring pressure based on a potential field.

### sEMG to normalised model activations

sEMG measures the total contribution from motor units beneath the electrodes as well as contributions from neighbouring motor units. sEMG is, by its nature, indiscriminate, and therefore, cross talk is inevitable. Moreover, in the complex face region muscles overlap and intertwine. A monopolar measurement configuration is more prone to cross talk as it measures the deeper and surrounding muscle signals, whereas in a bipolar configuration, the acquisition depth and pickup of cross talk depend on the interelectrode distance. Therefore, we recorded sEMG signals in bipolar configuration with a sample frequency of 2048 Hz. A fourth-order Butterworth band-pass filter with a high- and low-pass cut-off frequency of 15 and 500 Hz was used to filter the recorded sEMG signals, as recommended by van Boxtel [31]. The placement of our microelectrodes was done by considering the generic facial muscle anatomy and the optimal placement as described by Lapatki et al. [32]. A limiting factor was the face dimension of the volunteers and the corresponding availability of skin to place the electrodes, which was usually directly adjacent to each other (Fig. 1). Occasionally, no signal was sensed because of the tiny surface of the electrodes and the small surface of the underlying muscle belly. This occurred mainly when acquiring signals of the risorius muscle. In these cases, replacements of the microelectrode over 1–2 mm yielded good signal-to-noise ratios. Thus, a ruler-based placement strategy appeared to be impractical.

To generate input for the activation patterns of the ArtiSynth model, a transformation function was required that converted the sEMG signals from our seven bilaterally measured muscles into usable activations for ten bilateral muscles of the model. The design of this transformation was based on the study of Schumann et al. describing monopolar sEMG profiles of 30 healthy males for various instructions and on the activation patterns described by Flynn et al. [17, 25].

The measured muscles are: the orbicularis oris superior (OOS, electrodes 1, 2, 15, 16), the orbicularis oris inferior (OOI, electrodes 3, 4, 17, 18), the risorius (RIS, electrodes 9, 10, 23, 24), the zygomaticus major (ZYG, electrodes 11, 12, 25, 26), the levator labii superioris alaeque nasi (LLSAN, electrodes 5, 6, 19, 20), the depressor anguli oris (DAO electrodes 7, 8, 21, 22), and the mentalis (MEN, electrodes 13, 14, 27, 28). The missing muscle activations were determined as follows:

2 $$\begin{aligned} s_\mathrm{OOP}= & {} 0.50\cdot (s_\mathrm{OOS} +s_\mathrm{OOI} ) \end{aligned}$$

3 $$\begin{aligned} s_\mathrm{OOM}= & {} 0.10\cdot (s_\mathrm{OOP} +s_\mathrm{OOI} ) \end{aligned}$$

4 $$\begin{aligned} s_\mathrm{BUC}= & {} 0.50\cdot (s_\mathrm{RIS} +s_\mathrm{ZYG} ) \end{aligned}$$

5 $$\begin{aligned} s_\mathrm{LAO}= & {} 0.75\cdot s_\mathrm{LSSAN} \end{aligned}$$

6 $$\begin{aligned} s_\mathrm{DLI}= & {} 0.75\cdot s_\mathrm{DAO} \end{aligned}$$

In the ArtiSynth model, the orbicularis oris muscles are defined as peripheralis (OOP) and marginalis (OOM). Therefore, a combination of OOS and OOI was used for the OOP (Eq. 2). Based on Flynn et al. [17], a fifth of the OOP was used for the OOM (Eq. 3). The electrodes associated with the RIS were probably also influenced by the buccinator (BUC). Presumably, the electrodes of the ZYG were also influenced by the BUC. Therefore, we set BUC as a combination of ZYG and RIS activity (Eq. 4). The levator anguli oris (LAO) is close to the LLSAN. Thus, the LAO was set to 75% of LLSAN (Eq. 5). The depressor labii inferior (DLI) is adjacent to the DAO, which is why we chose 75% of the DAO as DLI activation (Eq. 6). The MEN, ZYG, RIS, DAO, and LLSAN muscles were set to their corresponding measurements.

**Table 1:** The muscles used with the relevant muscle strategy per instruction

Instruction	Relevant muscles ($\hbox {act}_\mathrm{rel}$)
Purse lips	OOP, OOM, BUC
Raise upper lip	LLSAN
Depress mouth corners	DAO, MEN
Voluntary smile	LLSAN, RIS, ZYG, LAO, DAO, DLI
Left-right-left with closed mouth	OOP, OOM, LLSAN, RIS, ZYG, LAO, BUC
Purse lips–closed mouth smile–purse lips	OOP, OOM, LLSAN, RIS, ZYG, LAO, BUC

In previous research in which we used statistical models [21, 22], the best performing sEMG feature extractor was Willison amplitude (WAMP) with a threshold $s_{{\mathrm{lim}}} =10$ mV and a sliding window of 200 ms with maximum overlap. In preliminary experiments for the current study we also tested the transfer function described by Buchanan et al. [33]. Eventually, the WAMP feature again proved to be best and therefore this feature was used for all the experiments described in this study. It can be calculated as follows for all sliding windows:

7 $$\begin{aligned}&g_m (t,i,r)=\sum _{n=1}^{N-1} {\left[ {f\left( {\left| {s_m (t+n-1)-s_m (t+n)} \right| } \right) } \right] }\nonumber \\&{\text {with }}\quad {f\left( {s_m } \right) =\left\{ \begin{array}{ll} 1\quad {\hbox {if}}\quad {s_m \ge s_{\lim } } &{} {i\text { is instruction index}} \\ 0\quad {\hbox {otherwise}}&{}{r\text { is repetition index}} \\ \end{array}\right. }\nonumber \\ \end{aligned}$$

$s_m (t)$ is the measured sEMG of muscle *m*, and *t* is the time index. *n* is the running time index within each sliding window consisting of *N* samples. There were six instructions: $i=1,\ldots , 6$. Each instruction was repeated four times: $r=1,\ldots , 5$. As there are ten muscles on both sides of the face, the muscle index runs from 1 to 10 (left) and 11 to 20 (right).

We tested three different activation strategies:

- $\hbox {act}_\mathrm{all}$: all the muscles in the model are activated.
- $\hbox {act}_3$: only the three muscles that were most active measured bilaterally according to:
  1. Muscle feature, averaged bilaterally: $\bar{{g}}_m (t,i,r)=\frac{1}{2}(g_m (t,i,r)+g_{m+10} (t,i,r))$
  2. Variance: $$V_m (i,r)=\hbox {Var}\left[ {\bar{{g}}_m (t,i,r)} \right] $$
  3. Sort: for each *i*, *r*: determine $m_j$ such that $V_{m_j} (i,r)\ge V_{m_{j+1}} (i,r)$
  4. Select largest three: $m_1$, $m_2$, and $m_3$
- $\hbox {act}_\mathrm{rel}$: the muscles that are considered most relevant for an instruction (Table 1).

The model’s activation range is from zero to one. Therefore, min–max normalisation was applied over the time index. It linearly transformed the data from original minimum and maximum to data between zero and one.

8 $$\begin{aligned} g_{\mathrm{norm},m} (t,i,r)=\frac{g_m (t,i,r)-\mathop {\min }\limits _{t} (g_m (t,i,r))}{\mathop {\max }\limits _{t} (g_m (t,i,r))-\mathop {\min }\limits _{t} (g_m (t,i,r))} \end{aligned}$$

### Synchronisation of repetitions and model output

As volunteers performed the repetitions with different speed and because the model’s output showed a different timing, a time shift and timescaling were performed. First, to create equally sized time series, we resampled the measurements (i.e. positions and features) in order to have them matched to the ArtiSynth sampling period. To synchronise the measurements, for each instruction and each repetition a principal component analysis (PCA) was applied to reduce the 30D space (ten 3D markers) to a 1D space. This was done both for the model-predicted positions and for the measured positions.

The PCA was implemented using singular value decomposition (SVD) of the $30\times \hbox {T}$ matrix $\mathbf{X}$ containing in each column the *X*-, *Y*-, and *Z*-coordinates of the 10 markers. The number T of columns equals the number of time samples. Application of SVD yields:

9 $$\begin{aligned} \mathbf{X}=\mathbf{U}{\varvec{\Sigma }} \mathbf{V}^\mathrm{T} \end{aligned}$$

The matrix $\mathbf{U}\in {\mathbb {R}}^{30\times 30}$ contains the principal components. The squares $\sigma _j^2$ of the diagonal of the matrix ${{\varvec{\Sigma }}}$ contain the variances of the principal components. These variances are sorted, $\sigma _j^2 \ge \sigma _{j+1}^2$. A coefficient vector $\mathbf{b}\in {\mathbb {R}}^{{\mathrm{T}}}$ was determined from the first principal component $\mathbf{u}_1 \in {\mathbb {R}}^{30}$ from $\mathbf{U}$:

10 $$\begin{aligned} \mathbf{b}=\mathbf{u}_1^T \mathbf{X} \end{aligned}$$

We obtained $\mathbf{b}_\mathrm{est}$ and $\mathbf{b}_\mathrm{meas}$ the coefficient vectors for the model-predicted positions and the measured ones, respectively. The maximisation of the cross-correlation function $\rho (t)$ between $\mathbf{b}_\mathrm{est}$ and $\mathbf{b}_\mathrm{meas}$ gave the synchronisation difference at $\arg \max \rho (t)$. The procedure was repeated for each instruction and repetition. Figure 3 shows the synchronisation process of two repetitions of one volunteer. The optimal shifting determined in the PCA domain was applied on the resampled data.

## Performance measures

**Fig. 4:**
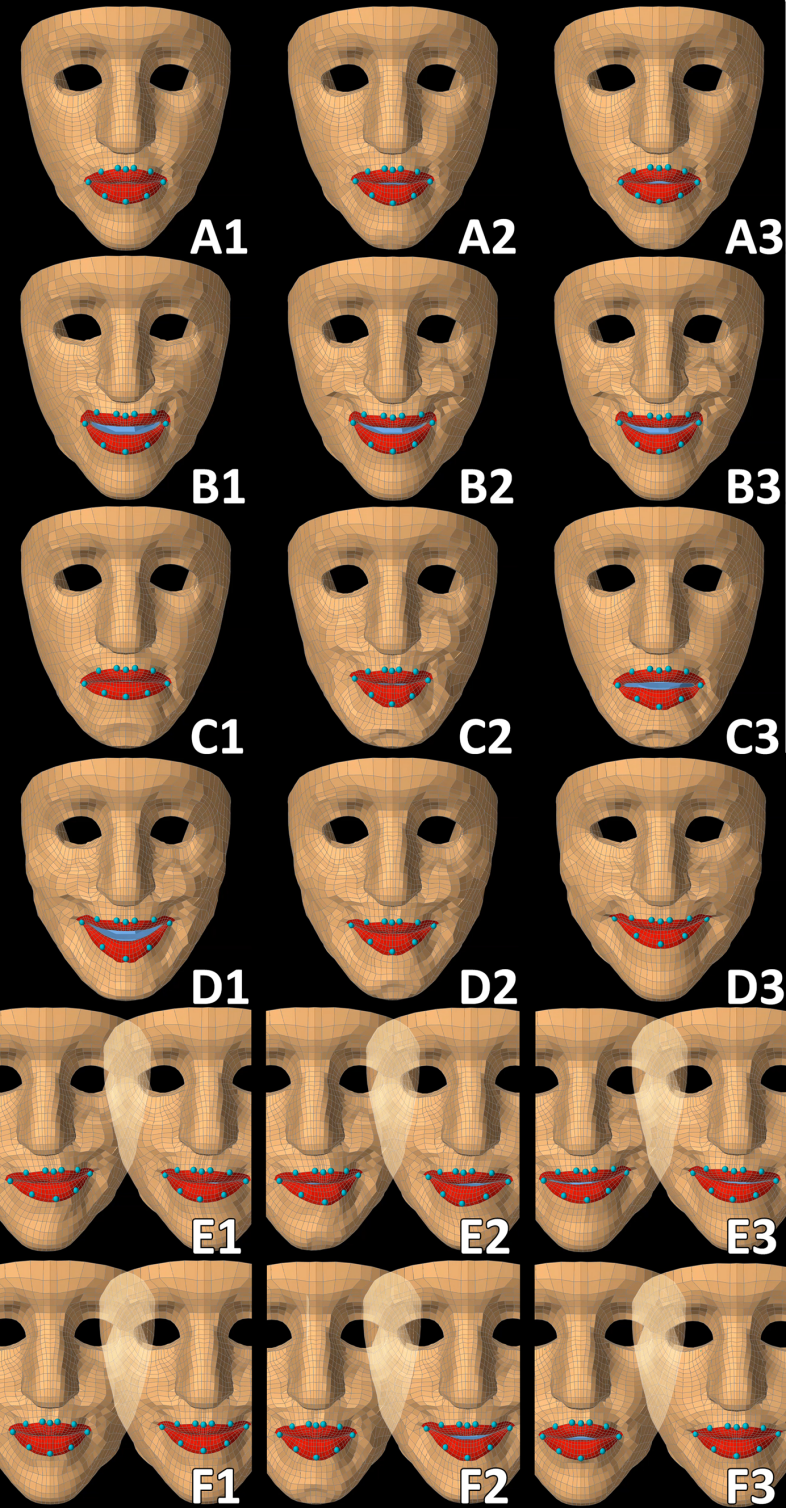
Qualitative simulation results for volunteer 2. The different instructions are represented by the capitals: **A** purse lips, **B** raise upper lip, **C** depress mouth corners, **D** voluntary smile, **E** left-right-left with closed lips (*left-right* is shown), **F** purse lips–closed mouth smile–purse lips (purse lips–closed mouth smile is shown). The three different activating strategies are given by the numbers: *1* $\hbox {act}_\mathrm{rel}$, *2* $\hbox {act}_\mathrm{all}$, *3* $\hbox {act}_3$. The ten cyan dots on the model’s lips are the nodes that are being compared to the volunteers’ tracked lip markers as shown in Fig. 1

Figure 4 and the online videos provide a qualitative visual impression. Quantitative performance measures are given by correlation coefficients for 3D quantities as provided by Pitermann and Munhall [34]:

11 $$\begin{aligned} \vec {\mu }_v= & {} \left( {\frac{1}{n}\sum _{i=1}^n {x_i } ,\frac{1}{n}\sum _{i=1}^n {y_i } ,\frac{1}{n}\sum _{i=1}^n {z_i } } \right) \end{aligned}$$

12 $$\begin{aligned} \sigma _v= & {} \sqrt{\frac{1}{n-1}\sum _{i=1}^n {\left\| {\vec {v}_i -\vec {\mu }_v } \right\| ^{2}} } \end{aligned}$$

13 $$\begin{aligned} \rho _{\vec {v}\vec {w}}= & {} \frac{\frac{1}{n}\sum \nolimits _{i=1}^n {\vec {v}_i ^\mathrm{T}\vec {w}_i -} \vec {\mu }_v ^\mathrm{T}\vec {\mu }_w }{\sigma _v \sigma _w } \end{aligned}$$

Equation (11) gives the mean position $\vec {\mu }_v$ of a 3D landmark trajectory of samples $\vec {v}_i =\left( {x_i ,y_i ,v_i } \right)$. The standard deviation $\sigma _v$ of the 3D node trajectory $\vec {v}_i$ is given by Eq. (12). $\rho _{\vec {v}\vec {w}}$ is the 3D correlation coefficient between 3D landmark trajectories $\vec {v}_i$ and $\vec {w}_i$ and is calculated with Eq. (13).

## Results

**Fig. 5:**
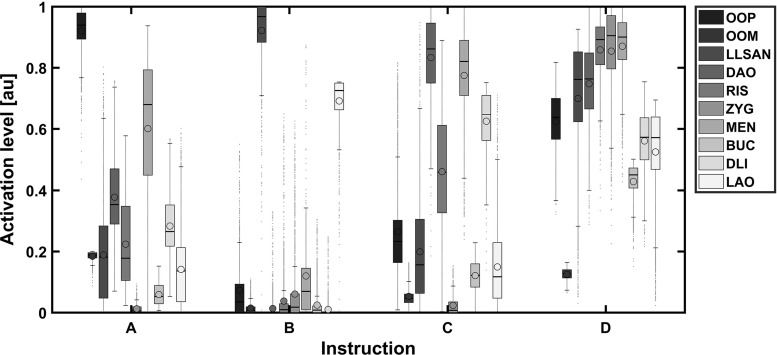
Boxplot of normalised sEMG features per instruction and per muscle including data of all volunteers and repetitions. High standard deviations indicate the volunteer-specific differences in activation strategies. The median is shown with a *horizontal line* and the mean with a *dot*

Fair performance for all activation strategies was seen in qualitative assessment (in Fig. 4 visuals are given for volunteer 2). Comparable results were obtained in all data sets (online videos show the performance of all volunteers). Activating the relevant facial muscles gave visual results that best matched the intended instructions. In general, the amplitude of the model’s movement was less than the volunteer’s movement. The three highest activated muscles differed among volunteers and sometimes also within repetitive measurements within one volunteer. This can be derived from Fig. 5 that gives the distribution of activation patterns of the symmetric instructions A–D. Instruction B (raise upper lip) showed the most selective contraction followed by instruction A (purse lips). Instruction C (depress mouth corners) showed a lot of cocontraction of the risorius muscle. Instruction D (voluntary smile) showed that indeed a lot of facial muscles come into play when producing voluntary smiles.

In all cases, at least one of the most important muscles ($\hbox {act}_3)$ was also present in the relevant muscle strategy. Comparing the model’s movements with those of the volunteers visually, the most difficult instruction was ‘pursed lips to closed mouth smile to pursed lips’ resulting in small displacements of the model. The easiest instruction was ‘raise upper lip’. Selectively depressing the lip corners was difficult to perform for most volunteers inducing a lot of cocontraction in the perioral region.

Pursing the lips (*A*) with $\hbox {act}_3$ resulted in a small opening between the lips in all volunteers, whereas $\hbox {act}_\mathrm{all}$ only had a minuscule opening in volunteer 2 and volunteer 3. $\hbox {act}_\mathrm{rel}$ had no opening between the pursed lips.

Raising the upper lip (*B*) with $\hbox {act}_\mathrm{all}$ showed less pronounced results, but more compressed lips drawn upwards in volunteers 2, 4, and 5.

Depressing the mouth corners (*C*) was difficult for the volunteers, but also to simulate with the model. Only $\hbox {act}_\mathrm{rel}$ gave visual satisfying results. $\hbox {act}_3$ had fair results in volunteers 2, 3, 5, and 6, though with an opening between the lips.

Voluntary smile (*D*) showing an open mouth smile was only possible with $\hbox {act}_\mathrm{rel}$, while $\hbox {act}_\mathrm{all}$ and $\hbox {act}_3$ resulted in closed mouth smiles except in volunteer 4 which had a modest open smile with $\hbox {act}_{3}$.

The instruction left-right-left with closed lips (*E*) in general showed modest displacements, but recognisable instructions with all activation strategies.

The instruction purse lips–closed mouth smile–purse lips (*F*) with $\hbox {act}_\mathrm{all}$ showed a small opening during closed mouth smile in volunteers 2, 4, 5, and 6, while in all volunteers $\hbox {act}_3$ induced a small opening between the pursed lips in the model.

**Fig. 6:**
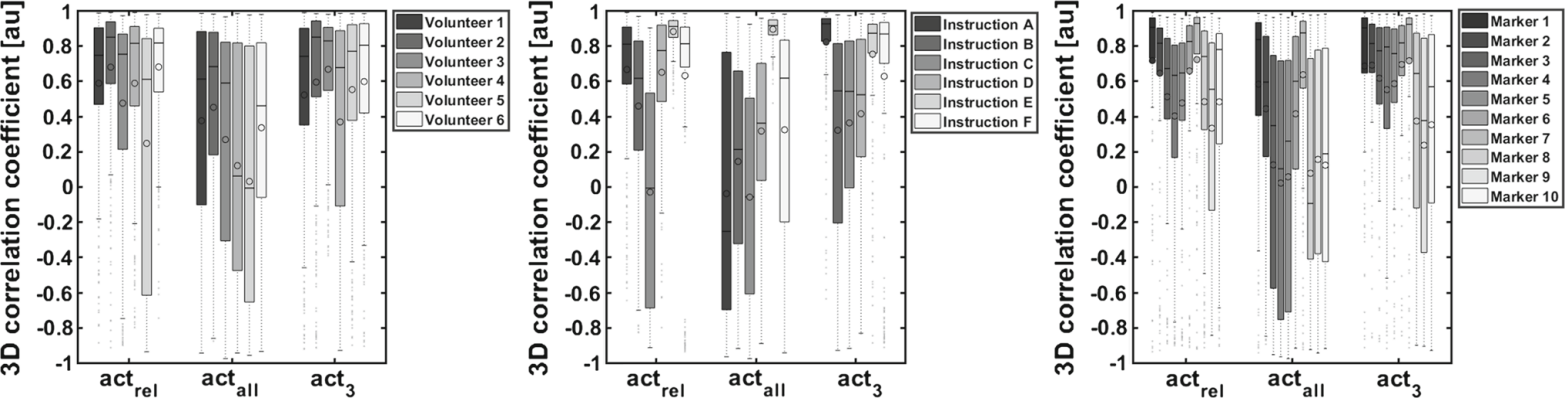
Boxplots of correlation coefficients per volunteer, per instruction, and per marker for the three activation strategies

The boxplots in Fig. 6 show the distribution of correlation coefficients between volunteers, instructions, and markers. Here, we have the following observations:

- The performance between volunteers differed, especially using $\hbox {act}_\mathrm{all}$. $\hbox {act}_3$ and $\hbox {act}_\mathrm{rel}$ had similar results.
- Although visually assessed instruction B was best executed by the volunteers, the corresponding correlation coefficients were not maximal. Instead, instructions E and F showed the best correlations. Again, $\hbox {act}_\mathrm{all}$ performed worst and act$_{3}$ and $\hbox {act}_\mathrm{rel}$ had similar results except for instruction *C*.
- The distribution of correlation coefficients between markers had a clear pattern: lateral markers showed higher correlations than centre markers, and upper lip markers had better results than lower lip markers in general. The overall mean values were: $\bar{{\rho }}_{\hbox {act}_\mathrm{all} } =0.26$, $\bar{{\rho }}_{\hbox {act}_3 } =0.55$, $\bar{{\rho }}_{\hbox {act}_\mathrm{rel} } =0.53$, with overall standard deviations: $\sigma _{\hbox {act}_\mathrm{all} } =0.63$, $\sigma _{\hbox {act}_3 } =0.51$, and $\sigma _{\hbox {act}_\mathrm{rel} } =0.52$, respectively. The medians were: $\rho _{\mathrm{act}_\mathrm{all} }^\mathrm{median} =0.45$, $\rho _{\mathrm{act}_3 }^\mathrm{median} =0.78$, and $\rho _{\mathrm{act}_\mathrm{rel} }^\mathrm{median} =0.77$.

## Discussion

To our knowledge, this is the first study demonstrating that volunteer-specific activation patterns calculated from sEMG measurements can be used to control a generic biomechanical model to generate asymmetric facial expressions with qualitative fair results. When visually assessed, the performance looked best when only the subset $\hbox {act}_\mathrm{rel}$ of all muscles was activated. These muscles were assumed to be most relevant for the specific instruction. Visual performance seemed to be less when activating all muscles $\hbox {act}_\mathrm{all}$, which should be the ideal situation containing all measured information. This loss of generality can be caused by different limitations of the method: (a) occurrence of cocontraction of pairs of muscles, (b) cross talk in the sEMG signals, (c) shortcomings in the transfer function from sEMG feature to activation signal, and (d) shortcomings in the biomechanical model, e.g. deviations from its optimal parameter setting, and deviation from the geometry.

**Fig. 7:**
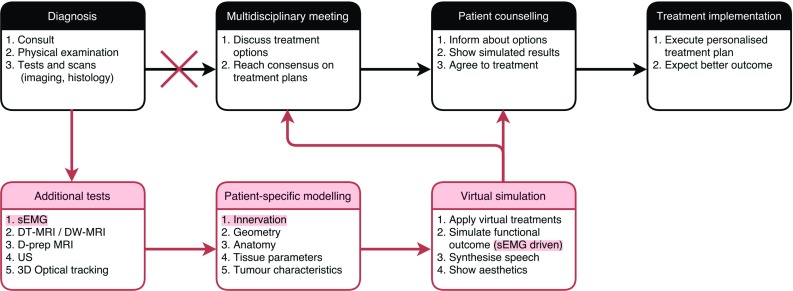
The *top black flow chart* resembles the current workflow in clinical practice. The *bottom red flow chart* shows the additional steps implementing virtual therapy to personalise and optimise the treatment per patient. The *red accentuated text* shows the importance of the current study

The quantitative results: the correlation coefficients, showed a large standard deviation also caused by the limitations as mentioned above. Predicted mobility of the 3D lip markers was less than measured in volunteers. This can be explained by inaccuracies in the tissue parameters (e.g. soft vs stiff skin parameters) and the resolution of the finite element model. Increasing the temporal step size and the number of elements will probably improve the accuracy. Improving the stability of the model for large deformations (possibly through model remeshing) is also essential. Lip shapes and thus the corresponding lip markers differed in volunteers. Subsequently, they did not match the generic face model’s geometry completely. This inaccuracy contributes to the mediocre values and high standard deviation of the correlation coefficients. Besides, instead of using an isotropic skin—all three layers had the same tissue parameters—a more sophisticated approach might be superior. This might be accomplished by giving each layer, or even regions within layers, specific material properties. This anisotropic skin model was first demonstrated by Flynn et al. [17].

The sEMG to muscle activation and finally muscle contraction is governed by a complex process. During recordings cross talk is inevitable. A solution could be the use of the cross talk equation of Lapatki et al. to determine whether an electrode is flooded with neighbouring signals [35]. Cross talk of adjacent muscles can explain why the model activated the OOP and OOM in all instructions. OOS and OOI electrodes 1, 2, 3, 4, 15, 16, 17, and 18, used to calculate OOP and OOM, could measure activity from, e.g. MEN, DAO, and LLSAN during those specific instructions. This results in OOP and OOM activity in the model. Intramuscular or needle EMG electrodes are more selective and are able to measure contributions of the single muscle, with less cross talk and reducing false input activity. However, we feel that a patient-friendly method and a less time-consuming method are preferred, especially when aiming at future preoperative modelling of patients to predict functional post-operative risk. sEMG cross talk problems might further be minimised by using high-density sEMG (HD-sEMG) [35]. Another point of attention is the arbitrary composition of the BUC, the DLI, and the LAO, out of neighbouring muscles. This induces an additional error. A future increase of bipolar or HD-sEMG measurements should compensate for this problem too.

Also, the muscle models may be improved. Instead of spherical muscles containing the surrounding elements within a radius of 5 mm of the muscle fibre, this radius can be optimised per muscle. Possibly by obtaining literature values or using patient-specific sizes to be determined in MRI scans of the patient, furthermore, one can manually assign elements or even improve the muscle representation using more than one muscle fibre, as was done by Wu et al. [20]. Another activation strategy option instead of $\hbox {act}_{3}$ is to determine the muscle channels showing activity that exceeds a certain threshold. The choice of three recruited muscles is arbitrarily, and the number of active muscles definitively differs per instruction and per person. This is demonstrated in the high standard deviations in the sEMG results of our experiments (Fig. 5) as well as the performance of $\hbox {act}_\mathrm{rel}$ versus $\hbox {act}_3$ (Fig. 6).

Although trivial we showed that asymmetric movement is possible in our modelling experiments, in contrast to previous research [18], creating unique opportunities for visualising possible consequences of surgery with a 3D render of the patient to get objective patient-specific information. This is particularly important as people never perform perfect symmetric movements, as is described by Campbell [36].

Many articles address the issue of facial surgery planning with a common goal of predicting aesthetics after facial surgery [5, 7, 37, 38]. Typically, surgical alteration (resection or replacement) of the bony structures underlying the face is applied to a virtual model and the resulting passive effects on soft tissues are then simulated. These principles of surgical alteration in static and dynamic situation and of rigid structures are important and will be addressed in our future models. A virtual surgery tool that can be used to simulate tumour resection in soft tissues is currently being developed in our institute. The tumour will be extracted from segmented MRI data before insertion into the model. Thereafter, the surgeon is able to perform a virtual resection of lip cancer followed by simulation of wound closure. The patient-specific sEMG measurements can then be used to control the adapted model to show residual movement after treatment, which is an essential part of the personalisation of the model. The promising results of our sEMG experiments are an important step in this process. We will first focus on the prediction of dynamic functions which are established by movement. Therefore, incorporating motor control strategies into the model as well as methods to assess motor control is essential. This is why we investigated the use of sEMG.

The results we created with forward dynamics took about 5 s per time step; each instruction was normalised to 160 time steps resulting in 800 s (13 min and 20 s) on a workstation with an Intel Xeon processor (3.40 Ghz). Guidelines in head and neck cancer care suggest 30 days from diagnosis to treatment [39]. Considering this time frame and the possibility of 24/7 runs of the analyses, we think the approach is definitively feasible within the given time. Even with the current set-up and without optimisation the analysis can be performed within the waiting time to treatment.

In the current workflow (Fig. 7), a patient is diagnosed and undergoes all kinds of scans and tests (imaging and histology). The case is presented to the multidisciplinary team to agree upon treatment. The proposed treatment plan is then explained to the patient. If the patient agrees, treatment is started.

In the future workflow (Fig. 7), after patient diagnosis and the standard imaging are performed several additional tests are done to obtain information on the specific patient. With all these data, a patient-specific model is built. Specific treatment modalities are tested, and the functional outcome (swallowing, speech, aesthetics, etc.) is simulated. The patient is again discussed at the multidisciplinary team meeting using the patient-specific modelling and simulations as objective aid. Next, the patient is informed about various treatment options considering survival, appearance, and functional outcome. These outcomes are made visible to the patient, who is also able to hear the post-treatment voice, if relevant. Together with the treating physician the patient decides which treatment is best suited for his/her expectations.

Our future experiments can be improved by using volunteer-specific biomechanical models. Recently, Bucki et al. [40] have described a method to adapt a model to volunteer-specific anatomy using personal imaging data and a Mesh-Match-and-Repair algorithm, while earlier Chabanas proposed a mesh correction algorithm after a mesh-matching procedure [11]. Additionally, we set the friction coefficient to zero as was done in previous models [14]. However, there is usually some amount of friction between the lips despite saliva, etc. Hence, this is probably not the best option. Future studies on simulation of facial expressions or bilabial and plosive speech articulations may benefit inclusion of a nonzero friction coefficient, though it should be investigated what the optimal value should be.

Patient-specific anatomy of in vivo muscle bundles may be extracted using diffusion tensor magnetic resonance imaging (DT-MRI), as suggested by Wu et al. [13]. Also, appropriate selection of most relevant personal parameters for inclusion in the model’s elements could be optimised per volunteer, such as tissue stiffness (which also depends on age), and muscle properties such as shortening.

Future experiments should also focus on inverse modelling. A known issue in biomechanical modelling is the ambiguity problem when sharing forces among a redundant set of muscles. In the case of multiple inverse solutions for the same motions, the resulting solution is based on mathematical properties instead of patient-specific factors. Using inverse modelling the required muscle activation patterns are calculated based on measured movement. It might be expected that incorporation of sEMG signals in the cost term, used to solve the inverse algorithm, contributes to the solution of the ambiguity problem [41]. Inverse modelling is also essential if one wants to incorporate compensatory muscle activity, which is important for the final functional result after surgery, and thus of importance in virtual therapy.

Other challenges concern preventive and rehabilitation exercises. Kraaijenga et al. [42] showed that senior healthy subjects are able to significantly increase swallowing muscle strength and muscle volume after a 6-week training period. van der Molen et al. [43] demonstrated beneficial effect of preventive swallowing exercises in patients undergoing chemoradiotherapy for advanced head and neck cancer. Given these facts, preventive and rehabilitation therapies can influence the functional outcome and thus the prediction of functional outcome. In the future, we hope to add decision support to point out the patients that benefit from pre- and post-operative speech, swallowing or other physical therapies. Besides, a virtual surgery tool and other treatment tools like radiotherapy should be implemented by utilising radiotherapy planning fields to determine which anatomical structures will be affected and to what extent.

To conclude, the use of sEMG opens new ways for patient-specific facial modelling, finally enabling us to predict the functional and cosmetic outcome after surgery. We applied a novel method to register two time sequences of vectors using the first principal components of these two vectors. Our experiments serve as a proof of principle for other opportunities as modelling of the oral cavity and tongue to predict function deficits after oral surgery, e.g. partial glossectomy, considering personalised muscle activation patterns. Although the extraction of muscle activation signals from tongue muscles is challenging, the epidermal electrodes described by Kim et al. [44] could be a promising option.

## Conclusion

Simulation of facial expressions using a biomechanical face model controlled by muscle activation signals estimated from volunteer-specific sEMG signals of facial muscles is feasible and may be useful for simulating function losses in the individual patient. Further experiments should focus on personalising the anatomical geometry of the model using MRI, CT, and DT-MRI, and development of methods to minimise cross talk between neighbouring muscles using HD-sEMG and advanced data processing techniques. Finally, these models can be expanded to other subsites of the head and neck like tongue, oropharynx, and larynx, while incorporating a virtual surgery tool and other treatments like photodynamic therapy, radiotherapy, and preventive and rehabilitation exercises.

## Electronic supplementary material

Below is the link to the electronic supplementary material.

Video 1. Volunteer 1's measured and model predicted movement with corresponding muscle activation patterns (Mp4 12,645KB)

Video 2. Volunteer 2's measured and model predicted movement with corresponding muscle activation patterns (Mp4 10,454KB)

Video 3. Volunteer 3's measured and model predicted movement with corresponding muscle activation patterns (Mp4 11,376KB)

Video 4. Volunteer 4's measured and model predicted movement with corresponding muscle activation patterns (Mp4 10,352KB)

Video 5. Volunteer 5's measured and model predicted movement with corresponding muscle activation patterns (Mp4 12,994KB)

Video 6. Volunteer 6's measured and model predicted movement with corresponding muscle activation patterns (Mp4 10,762KB)
